# Analysis of the Biceps Brachii Muscle by Varying the Arm Movement Level and Load Resistance Band

**DOI:** 10.1155/2017/1631384

**Published:** 2017-09-12

**Authors:** Nuradebah Burhan, Mohammad ‘Afif Kasno, Rozaimi Ghazali, Md Radzai Said, Shahrum Shah Abdullah, Mohd Hafiz Jali

**Affiliations:** ^1^Center for Robotics and Industrial Automation, Faculty of Electrical Engineering, Universiti Teknikal Malaysia Melaka, Hang Tuah Jaya, 76100 Durian Tunggal, Malaysia; ^2^Faculty of Engineering Technology, Universiti Teknikal Malaysia Melaka, Hang Tuah Jaya, 76100 Durian Tunggal, Malaysia; ^3^Faculty of Mechanical Engineering, Universiti Teknikal Malaysia Melaka, Hang Tuah Jaya, 76100 Durian Tunggal, Malaysia; ^4^Department of Electric and Electronics, Malaysia-Japan International Institute of Technology, Universiti Teknologi Malaysia, International Campus, Jalan Semarak, 54100 Kuala Lumpur, Malaysia

## Abstract

Biceps brachii muscle illness is one of the common physical disabilities that requires rehabilitation exercises in order to build up the strength of the muscle after surgery. It is also important to monitor the condition of the muscle during the rehabilitation exercise through electromyography (EMG) signals. The purpose of this study was to analyse and investigate the selection of the best mother wavelet (MWT) function and depth of the decomposition level in the wavelet denoising EMG signals through the discrete wavelet transform (DWT) method at each decomposition level. In this experimental work, six healthy subjects comprised of males and females (26 ± 3.0 years and BMI of 22 ± 2.0) were selected as a reference for persons with the illness. The experiment was conducted for three sets of resistance band loads, namely, 5 kg, 9 kg, and 16 kg, as a force during the biceps brachii muscle contraction. Each subject was required to perform three levels of the arm angle positions (30°, 90°, and 150°) for each set of resistance band load. The experimental results showed that the Daubechies5 (db5) was the most appropriate DWT method together with a 6-level decomposition with a soft heursure threshold for the biceps brachii EMG signal analysis.

## 1. Introduction

The National Institutes of Health (NIH), through the National Centre for Medical Rehabilitation Research (NCMRR) located in the United States, published a rehabilitation research plan in 1993 due to the increase in the range of disabilities among Americans affecting daily activities, work, and communication [1]. The rehabilitation research was aimed at improving, restoring, and developing the disabilities of the body or functions of the body system. This can help workers to recover physically and vocationally and, finally, return to the work area. In the rehabilitation method, the first assessment is necessary to identify the current condition of the patient's disability and his/her ability before the illness. It also includes a biopsychosocial model that emphasizes the physical functionality factor, the level of mobilization, and the physiological and environmental conditions, as well as identifies the needs of the patient on returning to work.

Biceps brachii muscle illness is a common physical disability that requires rehabilitation exercises in order to launch the movement and strengthen the weak biceps brachii muscle. The biceps brachii muscle condition can be measured by electromyography (EMG) [2], which helps to analyse the muscle activity signal produced by the desired muscle. The muscle activity signal is generated by an electrical signal that originates from the activation of the muscle fibres by a motor unit. It can be detected and measured by using EMG electrodes.

The EMG electrodes are of two types. The surface EMG (sEMG), which is commonly used in biomedical techniques, is known as a noninvasive method, while the needle EMG is an invasive method. The sEMG method is a convenient EMG measurement method as it can be easily implemented without any medical certificate, where the sEMG electrodes that are used can be placed on the desired skin surface to record the activity of the muscle [3]. However, the detection of the EMG signals is a complex process that is easily affected by a combination of numerous noise signals, the motion artefact, and the internal structure of the human body, such as the skin formation, velocity of the blood flow, and thickness of the fatty tissue [4]. It shows the recorded EMG signals, called the raw EMG signals, that contain information about the muscle and several noises during the EMG measurement. In order to obtain useful information from the EMG signals, several approaches in terms of feature extraction must be considered when analysing the performance of the EMG signal.

Feature extraction is the main part in signal processing in order to eliminate the affected noise or undesired part and to obtain the useful information in the EMG signals. Feature extraction can be categorized into three methods, namely, for the extraction of time domain (TD), frequency domain (TF), and time-frequency domain (TFD) features. Previous studies have mentioned that a stationary sEMG signal depends on many factors such as the contraction of the muscle under the application of a constant force, where the sEMG signal would be considered as stationary, which is a TD feature [5]. In the meantime, the sEMG signal is also considered as nonstationary because it is contained in various frequency components [6]. Thus, wavelet transform, as a TFD feature, is the best feature extraction technique for analysing the sEMG in both the time and frequency domains.

Several authors have described the EMG signal analysis performance and their validation of the biceps brachii muscle with different ranges of age, protocols, and electrode placements on the desired muscle. For example, the monitoring of an athlete's performance in muscle strength exercises focuses on the use of a dumbbell as a resistance to muscle contraction [7] in order to increase the strength of the biceps brachii muscle. A previous study discussed and compared the effect of electromyography on the biceps brachii muscles of male and female subjects. The comparisons were based on the root mean square and mean values [8]. Many studies have attempted to analyse the contraction signals of the biceps brachii muscle in three different age groups, namely, adolescents (younger age), vicenarians (middle age), and tricenarians (elderly age). In their research, the comparison of the electromyographic biceps brachii muscle activity was based on differences in the root mean square (RMS) and mean absolute value (MAV), which are the most commonly accepted features that are used to define the amplitude of electromyography signals [9, 10]. Some researchers discussed the placement of the electrodes on the biceps brachii muscle during the EMG measurement. The best location for the EMG electrodes is in the area between the innervation zone (IZ) and the tendon to obtain high-quality and stable sEMG signals [11, 12].

This shows that previous researches into electromyography concentrated more on the performance of the biceps brachii muscle with regard to several factors based specifically on age and gender. Others clarified the role of the biceps brachii muscle in shoulder elevation and elbow flexion and extension movements [13, 14]. These were examined based on several variable factors such as the type of external load, contraction, and elbow joint angles.

Thus, this research was inspired to focus on analysing the electromyography signals from the biceps brachii muscle for resistance band rehabilitation exercises. The EMG measurement was made during the isometric muscle contraction for three angles at the arm level. This study was to investigate the difference in the sEMG signals on the muscles of vicenarians during the resistance band rehabilitation exercises in terms of gender and types of loads during muscle contraction at three angles at the arm level. A fixed sampling rate of 1000 Hz and a wireless EMG preamplifier were used.

## 2. Materials and Method

### 2.1. Subjects

Six healthy subjects, who were right-hand dominant, participated in this study. The six healthy subjects were categorized based on gender into three healthy male subjects and three healthy female subjects. All the subjects were vicenarians between the ages of 23 to 27 years. According to investigations in previous works, vicenarian subjects are within the best range of age as references for the human body in EMG measurements, where the muscles of those in middle age have grown gradually and a higher amplitude of EMG signals can be obtained during the EMG measurement process [15]. The normal body mass index (BMI) was one of the preferred physical characteristics of the subjects that was considered in this study.  Table 1 shows the physical characteristics of all the subjects.

### 2.2. Experimental Setup and Protocols

A wireless Z03 EMG preamplifier with surface recording of the ground by Motion Lab Systems Inc. (Baton Rouge, LA, USA) was used for the EMG signal recording. The EMG preamplifier is a compact device with 12 mm disks with an interelectrode distance of 18 mm and one reference contact (12 × 3 mm) bar separating the sensors. Medical-grade stainless steel was used as the contact material for the electrodes in this experiment. The EMG preamplifier had a gain at 1 kHz × 300 ± 1%, CMRR > 100 dB at 65 Hz, input protection from radio frequency interference (RFI) filters, and electrostatic discharge (ESD), while the power supply range of this device was between ±5 Volts and ±15 Volts.

Some protocols had to be considered before the start of the experiment. First, the subjects had to be free of any muscular disease and avoid strenuous exercise on the biceps brachii muscle for two days prior to the EMG measurement. Second, the subjects needed to perform 5 minutes of warm-up stretching exercises with the lifting and lowering of weights, with an interval of at least 2 minutes between muscle contractions to avoid the possibility of muscle fatigue. The third protocol was the clarification about the procedure for the placement of the electrodes and the skin preparation. It was necessary to prepare the skin by cleaning the desired skin area using 70% isopropyl alcohol and shaving the hair, if necessary, in order to reduce the electrode-skin impedance [16]. The preferred placement of the EMG electrodes on the biceps brachii muscle, as suggested in previous works, is in the middle of the biceps brachii muscle, known as the belly muscle, as it shows a significantly higher amplitude [17]. All the protocols were designed to minimize the motion artefact, crosstalk, and internal noise during the EMG measurement.

**Figure 1:**
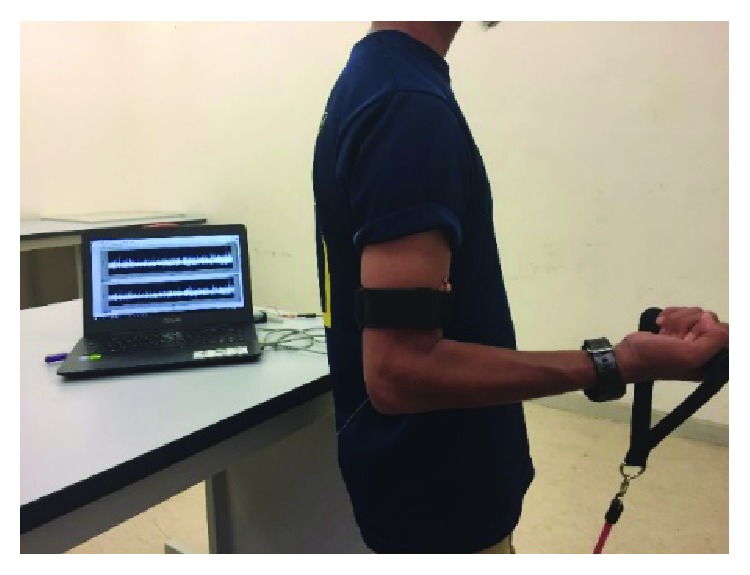
EMG data being recorded when angle at the arm level is at 90°.

This experiment consisted of three sets of resistance band loads of 5 kg, 9 kg, and 16 kg that were used as a force during the biceps brachii muscle contractions. Each subject was required to stand up straight and perform three levels of arm angle positions (30°, 90°, and 150°) for each set of resistance band loads. The arm angle position was measured using a Medigauge electronic digital goniometer. The subjects had to hold the resistance band for 10 seconds and then take a break for a time interval of 2 minutes for each movement of the arm level. The procedure was repeated 10 times per set of resistance band loads. This is illustrated in Figure 1, which shows the subject holding the resistance band for 10 seconds when the angle at the arm level was at 90°. The resistance band is one of the preferred tools in biceps brachii rehabilitation exercises, where it is currently being used in rehabilitation centres to train patients to build up the strength of their biceps brachii muscle after surgery or injury.

### 2.3. EMG Signal Processing

In the experimental setup, a compact wireless EMG preamplifier device was used to supply the input signal to an NI USB-6009 data acquisition (DAQ) device from National Instruments, where the raw signal was recorded at a sampling rate of 1000 Hz. The signal acquired from the DAQ device acted as a signal source for the LabVIEW 2016 model. Subsequently, the recorded EMG signal was processed by filtering and extracting the useful signals with the LabVIEW WA Detrend VI and LabVIEW Wavelet Denoise Signal. The discrete wavelet transform (DWT) approach was implemented in the EMG signal analysis. Based on the previously mentioned work, the DWT was better than the continuous wavelet transform (CWT) approach, which did not yield a redundant analysis [16].

The DWT algorithm uses a filtering technique that consists of a shifted and scaled version of a certain function called a mother wavelet transform (MWT) function, *ψ*(*t*) [18]. The MWT is shifted by time (*b*) and scaled by a factor (*a*), as in

(1) $$\begin{array}{c} DWTa,b\left(f\right)=\frac{1}{\sqrt{a}}\int f\left(t\right)\psi \left(\frac{t-b}{a}\right)dt. \end{array}$$

In this method, the DWT will decompose a signal into different frequency bands by passing it through two filters, namely, a low-pass filter (*h*) and a high-pass filter(*g*) at each decomposition level. Both filters are associated with the scaling function, *φ*, and the MWT function, *ψ*, where the scaling function is related to the low-pass filter and the MWT function is related to the high-pass filter [19], which can be shown through the following equations:

(2) $$\begin{array}{c} \varphi =2\overset{N-1}{\underset{n=0}{\sum }}h\left(n\right)\varphi \left(2t-n\right),\\ \psi =2\overset{N-1}{\underset{n=0}{\sum }}g\left(n\right)\psi \left(2t-n\right). \end{array}$$

These equations will be followed by downsampling by the factor of 2 in order to obtain the successive DWT filtering of the time domain signal. The output of the downsampled low-pass filter produces an approximation coefficient, cA*i*, whereas the downsampled high-pass filter produces the detailed coefficient, cD*i*, of the depth decomposition level, *i*. The equations for the filters can be expressed by

(3) $$\begin{array}{c} {cA}_{i}\left(k\right)=\sum_{n=0}{cA}_{i-1}\left(n\right)h\left(2k-n\right),\\ {cD}_{i}\left(k\right)=\sum_{n=0}{cA}_{i-1}\left(n\right)g\left(2k-n\right). \end{array}$$

### 2.4. Mother Wavelet and Decomposition Level Selection

In the denoising signal, several common MWT functions, such as Daubechies, Coiflet, and Symlet, are used. The selection of the best wavelet function and depth of decomposition is required to produce a perfect reconstruction and better signal analysis [17]. The best MWT function and decomposition level were determined by calculating the signal to noise ratio (SNR) and root mean square error (RMSE), as given below [19].

(4) $$\begin{array}{c} SNR\left(dB\right)=10\;\log_{10}\left(\frac{\sum_{n=1}^{N}x{\left[n\right]}^{2}}{\sum_{n=1}^{N}{\left(\hat{x}\left[n\right]-x\left[n\right]\right)}^{2}}\right),\\ RMSE=\sqrt{\frac{1}{N}\overset{N}{\underset{n=1}{\sum }}{\left(\hat{x}\left[n\right]-x\left[n\right]\right)}^{2},} \end{array}$$

where *x*[*n*] is the noise-free EMG signal and $\hat{x}\left[n\right]$ is known as the denoised signal, while *N* is the number of signal samples. In this study, the value of *N* was 10000.

The SNR is defined as the ratio of the variance of the noise-free signal to the mean square error between the noise-free signal and the denoised signal, and it is the measurement of the signal strength relative to the background noise. It is measured in decibels (dB). The RMSE indicates the absolute measure of fit, which evaluates the closer of the observed data points to the predicted values.

### 2.5. Statistical Analysis

In this study, a statistical analysis was applied to the EMG signals and was executed using the MATLAB software. All the filtered EMG signals were analysed in terms of the average (Avg), standard deviation (SD), and root mean square (RMS). The Avg, SD, and RMS were obtained by using the statistical equations as follows:

(5) $$\begin{array}{c} average\;\left(Avg\right)\left(\hat{x}\right)=\frac{1}{N}\overset{N}{\underset{i=1}{\sum }}x_{i},\\ standard\;deviation\;\left(SD\right)\left(\sigma \right)=\sqrt{\frac{1}{N}\overset{N}{\underset{i=1}{\sum }}{\left(x_{i}-\bar{x}\right)}^{2},}\\ root\;mean\;square\left(RMS\right)=\sqrt{\frac{1}{N}\overset{N}{\underset{i=1}{\sum }}{x_{i}}^{2},} \end{array}$$

where *x*_*i*_ is the noise-free signal collected and *N* is the number of signal samples.

## 3. Results and Discussion

In this experiment, the biceps brachii EMG signals of the subjects were obtained at a six-level decomposition coefficient through the DWT method. Seven subbands were involved, namely, cD1, cD2, cD3, cD4, cD5, cD6, and cA6, which represented the frequency range from the band limit of the EMG signal. The selection of a suitable decomposition level was necessary to extract the useful information and analyse the EMG signal by using the DWT method. Other than that, the threshold function and limit were also the main factors in ensuring that the useful information of the EMG signal would be able to be extracted using the WT denoising technique. Based on that, the heursure threshold, with a soft thresholding method, was proposed to analyse the EMG signal.  Table 2 presents the SNR and RMSE results with respect to the decomposition level by using the Daubechies5 (db5) and heursure thresholding method. The results for both the SNR and RMSE values showed the best performance at the 6-level decomposition through the DWT method at each level, where the highest SNR value and lowest RMSE value were obtained. The highest SNR value showed the strength of the EMG signal that was acquired. The lowest value of the RMSE illustrated a better fit of the signal data.

Consequently, the best SNR and RMSE values were required to determine the suitable MWT function at the 6-level decomposition of the EMG signal analysis. The MWT functions that were investigated in previous studies, such as Daubechies, Coiflet, and Symlet, have their own suitability that depends on the types of signals in the biomedical field that need to be analysed, where the Daubechies2 (db2) is more appropriate for the electroencephalography (EEG) smoothing signal, the Daubechies4 (db4), Coiflet3 (coif3), Coiflet4 (coif4), and Coiflet5 (coif5) are able to improve the electrocardiography (ECG) detection signal in their applications, and the Daubechies5 (db5) is convenient for use in the removal of noise from the EMG signal [20–24]. This can be further strengthened with the SNR and RMSE results for the db5 as the optimal MWT in Table 3, where it is shown that the db5 with a 6-level decomposition and soft heursure threshold through the DWT method is suitable for the biceps brachii EMG signal analysis.

The EMG denoising technique, using the DWT method with the appropriate MWT function (db5) and depth of the decomposition level (6-level), was implemented in the rehabilitation application focusing on biceps brachii illness. The efficacy of the EMG denoising technique used was determined by calculating the SD values for the subjects in every given task. The results of the EMG signal analysis were classified according to the gender. It consisted of three types of resistance band loads, namely, 5 kg, 9 kg, and 16 kg. Each set of resistance band loads contained three different angles of the arm level. Tables 4, 5, and 6 show the results for the male subjects, whereas the results of the statistical values for the female subjects are presented in Tables 7, 8, and 9.

**Figure 2:**
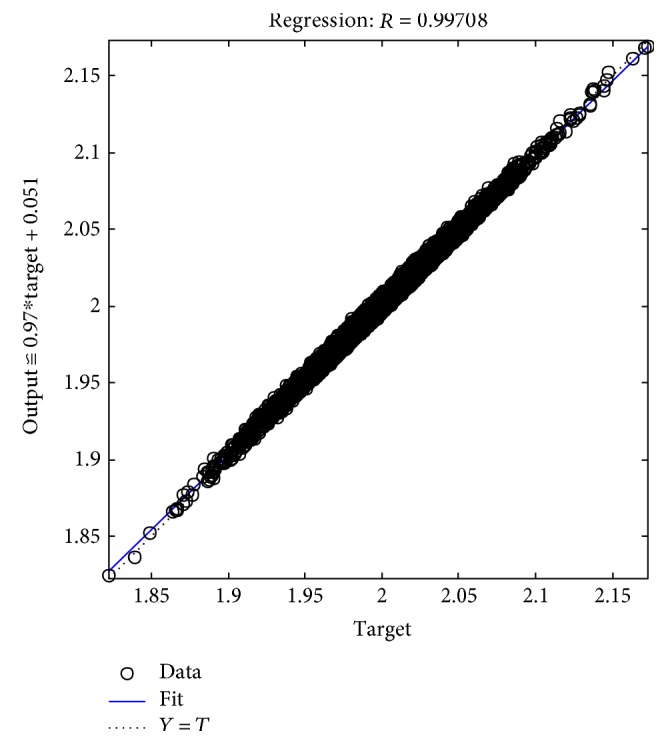
Male linear regression for 5 kg.

**Figure 3:**
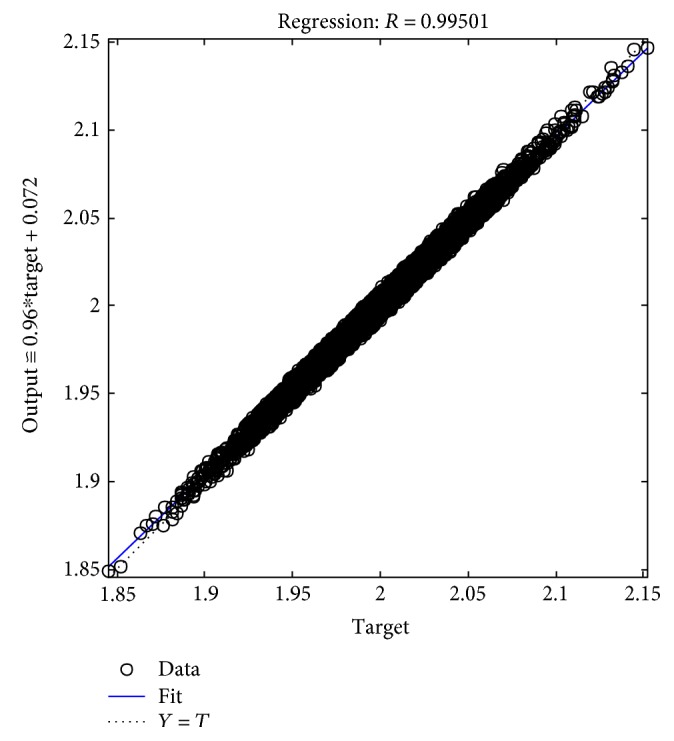
Male linear regression for 9 kg.

**Figure 4:**
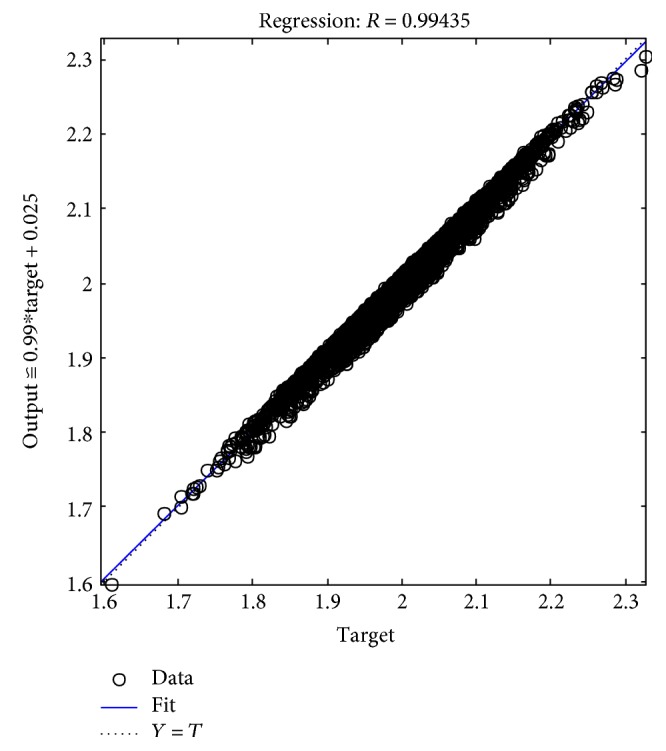
Male linear regression for 16 kg.

**Figure 5:**
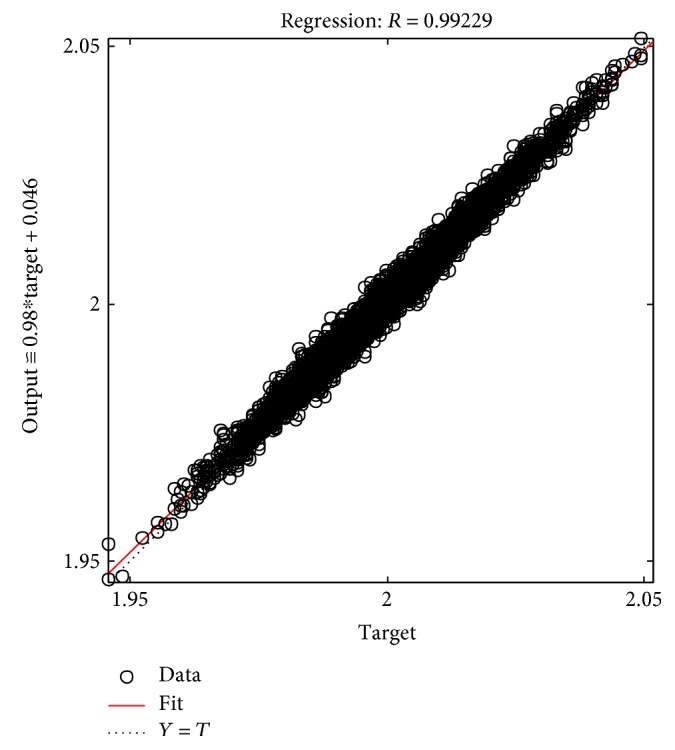
Female linear regression for 5 kg.

**Figure 6:**
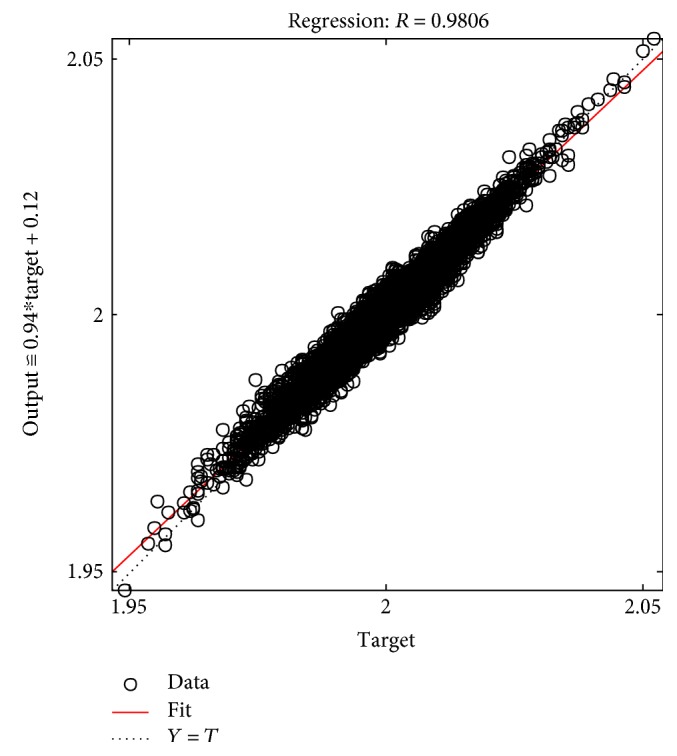
Female linear regression for 9 kg.

**Figure 7:**
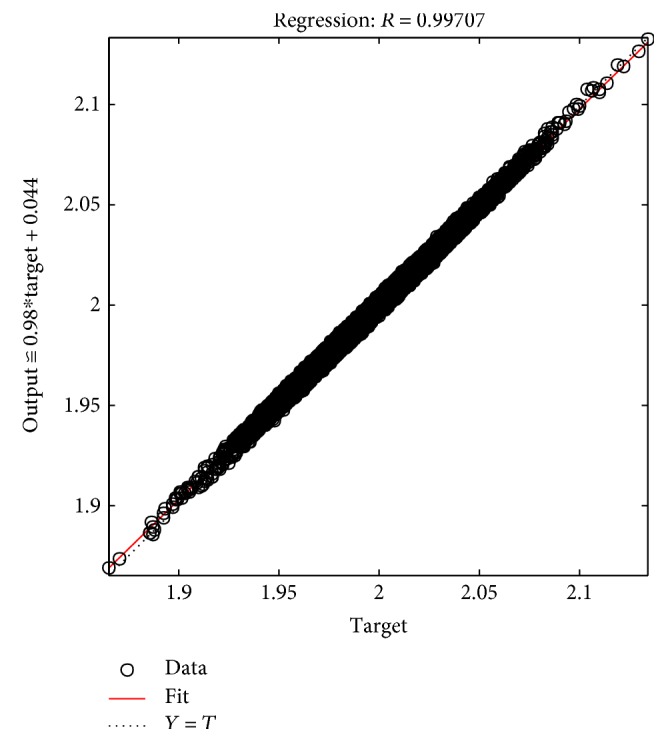
Female linear regression for 16 kg.

The tables above show that the SD values had a smaller range, where the SD range for the male subjects was from 0.006 to 0.0695 and the SD range for the female subjects was from 0.005 to 0.0624. This indicated the clustered data of the EMG signals produced during the rehabilitation exercise. The lowest SD in the statistical data in this experiment showed that the data had a good performance. The good performance of the statistical data in this experiment was shown in the regression performance for both genders. Figures 2, 3, and 4 present the regression results of the male subjects, while Figures 5, 6, and 7 show the regression results for the female subjects for three different load resistance bands when the arm angle was at 30°. The regression, *R*, in each load resistance band for both genders was above 0.92. The regression plots displayed the perfect fit of the data, where the data fell along a 45° line, thereby indicating that the data obtained were equal to the targets. This indicated a good accuracy performance of the data obtained by using the appropriate db5 as a MWT function and a 6-level decomposition through the DWT method in the EMG denoising. The best performance of the denoising EMG signals acquired helped to obtain a better feature extraction and classification of the EMG signals. Consequently, it helped to classify the EMG patterns of the three different angles of the arm level in this rehabilitation application.

## 4. Conclusion

In this study, the compatibility of the three common MWT functions, namely, Daubechies, Coiflet, and Symlet, were selected for analysis to determine an optimal MWT function in order to obtain the best performance for the denoising of the EMG signals. This experiment was able to successfully select the optimal MWT function and depth of the decomposition level with the best performance of the EMG signal denoising with the EMG datasets of the six subjects. Based on the analysis in this study, it was concluded that the “db5” with a “6-level decomposition” is more appropriate for denoising the EMG signal of the biceps brachii muscle in order to obtain a better performance on the feature extraction and classification technique of the EMG signal in the rehabilitation application.

## Figures and Tables

**Table 1:** Physical characteristics of subjects.

Gender	Age (years)	Weight (kg)	Height (cm)	BMI (kg/cm)
Male	24	70	172	23.5
	25	68	169	23.8
	27	69	170	23.9

Female	23	55	157	22.3
	25	58	160	22.7
	26	54	159	21.4

**Table 2:** SNR and RMSE results with respect to the decomposition level.

Decomposition level	SNR (dB)	RMSE (^∗^10^−3^)
1	57.056	2.80
2	56.946	2.83
3	56.945	2.83
4	56.946	2.80
5	56.960	2.80
6	56.992	2.80
7	56.919	2.83
8	56.901	2.87
9	56.872	2.90
10	56.901	2.87
11	56.901	2.87
12	56.906	2.87
13	56.901	2.87

**Table 3:** SNR and RMSE results with respect to wavelet types.

Wavelet types	SNR (dB)	RMSE (^∗^10^−3^)
Daubechies2 (db2)	54.1161	3.93
Daubechies3 (db3)	56.1163	3.13
Daubechies4 (db4)	56.7373	2.93
Daubechies5 (db5)	56.9924	2.80
Coiflet2 (coif2)	56.8161	2.90
Coiflet3 (coif3)	57.0431	2.80
Coiflet4 (coif4)	56.9870	2.83
Coiflet5 (coif5)	57.0415	2.83
Symlet2 (sym2)	56.8161	2.90
Symlet3 (sym3)	56.1163	3.13
Symlet4 (sym4)	56.6773	2.93
Symlet5 (sym5)	57.0152	2.80

**Table 4:** Results of three male subjects for 5 kg.

Gender	Angles	Statistical values		
		Avg	SD	RMS
Male 1	30°	2.0001	0.0312	2.0003
	90°	2.0001	0.0511	2.0008
	150°	2.0006	0.0549	2.0013

Male 2	30°	2.0000	0.0060	2.0000
	90°	2.0000	0.0142	2.0001
	150°	2.0000	0.0093	2.0000

Male 3	30°	2.0000	0.0123	2.0001
	90°	2.0000	0.0214	2.0001
	150°	2.0001	0.0347	2.0004

**Table 5:** Results of three male subjects for 9 kg.

Gender	Angles	Statistical values		
		Avg	SD	RMS
Male 1	30°	2.0001	0.0299	2.0003
	90°	2.0001	0.0480	2.0007
	150°	2.0001	0.0664	2.0013

Male 2	30°	2.0000	0.0077	2.0000
	90°	2.0000	0.0111	2.0000
	150°	2.0000	0.0105	2.0000

Male 3	30°	2.0000	0.0188	2.0001
	90°	2.0001	0.0299	2.0003
	150°	1.9999	0.0357	2.0002

**Table 6:** Results of the three male subjects for 16 kg.

Gender	Angles	Statistical values		
		Avg	SD	RMS
Male 1	30°	1.9995	0.0686	2.0007
	90°	2.0000	0.0509	2.0006
	150°	1.9999	0.0695	2.0011

Male 2	30°	2.0000	0.0078	2.0000
	90°	2.0000	0.0121	2.0000
	150°	2.0000	0.0102	2.0000

Male 3	30°	2.0000	0.0326	2.0003
	90°	2.0000	0.0371	2.0004
	150°	2.0000	0.0670	2.0011

**Table 7:** Results of three female subjects for 5 kg.

Gender	Angles	Statistical values		
		Avg	SD	RMS
Male 1	30°	2.0000	0.0097	2.0001
	90°	2.0000	0.0135	2.0000
	150°	2.0000	0.0207	2.0001

Male 2	30°	2.0000	0.0177	2.0002
	90°	1.9999	0.0256	2.0000
	150°	2.0000	0.0306	2.0003

Male 3	30°	2.0000	0.0057	2.0000
	90°	2.0000	0.0088	2.0000
	150°	2.0000	0.0089	2.0000

**Table 8:** Results of three female subjects for 9 kg.

Gender	Angles	Statistical values		
		Avg	SD	RMS
Male 1	30°	2.0000	0.0111	2.0000
	90°	2.0000	0.0192	2.0001
	150°	1.9999	0.0366	2.0002

Male 2	30°	2.0000	0.0248	2.0002
	90°	2.0000	0.0187	2.0001
	150°	2.0000	0.0199	2.0000

Male 3	30°	2.0000	0.0063	2.0000
	90°	2.0000	0.0143	2.0001
	150°	2.0000	0.0129	2.0000

**Table 9:** Results of three female subjects for 16 kg.

Gender	Angles	Statistical values		
		Avg	SD	RMS
Male 1	30°	2.0000	0.0276	2.0002
	90°	2.0000	0.0425	2.0004
	150°	1.9999	0.0568	2.0007

Male 2	30°	2.0001	0.0369	2.0004
	90°	2.0003	0.0624	2.0012
	150°	2.0001	0.0326	2.0003

Male 3	30°	1.9999	0.0176	2.0000
	90°	2.0001	0.0225	2.0002
	150°	2.0000	0.0228	2.0001
